# Fluoroscopy-Assisted ICE-Guided Tricuspid Valve Vegetation Removal in CIED-Related Infective Endocarditis

**DOI:** 10.1016/j.jaccas.2025.105484

**Published:** 2025-10-22

**Authors:** Musa'ab Moh'd Alhmouz, Batool Jamal Abuhalimeh, Mo'ath Bani Ali, John Rickard, Mohamed Al Jaabari, Mohammed E. Khalil, Mohammad Qadura, Raghad Abuhalimeh, Lina Abuhalimeh, Houssam K. Younes

**Affiliations:** Cleveland Clinic, Abu Dhabi, United Arab Emirates

**Keywords:** cardiac implantable electronic device (CIED), cardiac implantable electronic device–related infective endocarditis (CIED-IE), cardiac resynchronization therapy with defibrillator (CRT-D)

## Abstract

**Background:**

Cardiac implantable electronic device–related infective endocarditis (CIED-IE) presents diagnostic and therapeutic challenges owing to variable clinical presentations and imaging limitations. Guidelines differ on managing large vegetations, highlighting the need for individualized clinical decision-making.

**Case Summary:**

A 69-year-old man with previous cardiac resynchronization therapy with defibrillator implantation presented with fever, candidemia, and a 4-cm mobile vegetation attached to the tricuspid lead. After multidisciplinary evaluation, he underwent fluoroscopy-assisted, intracardiac echocardiography–guided aspiration to debulk the vegetation, then had successful device and lead extraction.

**Discussion:**

This case highlights the value of advanced endovascular techniques in reducing embolization risk and improving outcomes in high-risk patients with CIED-IE.

**Take-Home Messages:**

Multimodality imaging is essential for accurate diagnosis of CIED-IE. Fluoroscopy-assisted, intracardiac echocardiography–guided vegetation aspiration is a promising adjunctive technique that can enhance procedural precision and safety, although larger studies are needed to clarify its role in reducing septic emboli and controlling infection.

## Graphical Abstract

**Figure:**
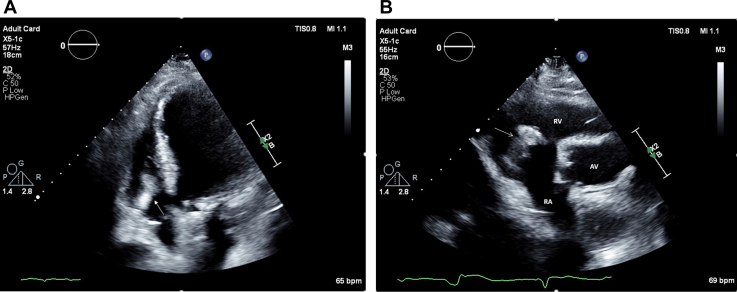

## History of Presentation

A 69-year-old man, a chronic smoker, presented to the emergency department with fever, chills, and acute epigastric abdominal pain. He was subsequently diagnosed with calculus cholecystitis and was managed conservatively with antibiotics. During hospitalization, he was found to have persistent fever and elevated inflammatory markers despite coverage with wide-spectrum antibiotics.

**Take-Home Messages**

• Accurate diagnosis and early intervention: Multimodality imaging is critical for confirming CIED-IE and guiding timely, individualized treatment decisions.
• Innovative adjunctive techniques: Fluoroscopy-assisted, ICE-guided aspiration offers a promising, minimally invasive option for debulking large vegetations, highlighting the need for further research and standardized protocols.

## Past Medical History

The patient had a complex cardiac and vascular history, including paroxysmal atrial fibrillation. In 2023, he underwent cardiac resynchronization therapy with defibrillator (CRT-D) placement owing to heart failure with reduced ejection fraction (ejection fraction: 30%) for primary prevention of sudden cardiac death. His cardiac history further included a coronary artery bypass graft to the right coronary artery, with a vein graft and a mechanical aortic valve replacement performed in 2012 for aortic stenosis. Also in 2023, he underwent percutaneous coronary interventions with stent placements in both the right coronary artery and left circumflex artery. Additionally, he had peripheral artery disease, for which a stent was placed in the right femoral artery in 2022. Other significant comorbidities included chronic kidney disease stage III, hypertension related to renal artery stenosis, and a longstanding history of smoking. The patient also had a history of sigmoid diverticulitis complicated by a small bowel fistula, which necessitated a colectomy with small bowel resection at the fistula site and a diversion ileostomy in 2024.

## Physical Examination

The patient appeared unwell, septic with persistent fever; cardiac examination revealed new tricuspid murmur on auscultation. Respiratory examination showed bilateral clear lungs. Neurological examination was unremarkable.

## Investigations

**Figure 1:**
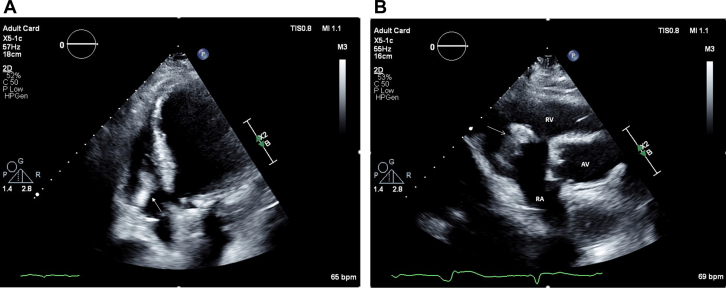
Transthoracic Echocardiography of Echodense Mass (A) Apical 4-chamber view revealing a 4-cm echodensity involving the tricuspid valve near the atrial pacing lead and attached to posterior tricuspid leaflet (arrow); this echodensity was found to be prolapsing back and forth across the valve, with mild tricuspid regurgitation. (B) Parasternal short-axis view showing echodense mass attached to posterior tricuspid valve (arrow). AV = aortic valve; RA = right atrium; RV = right ventricle.

**Figure 2:**
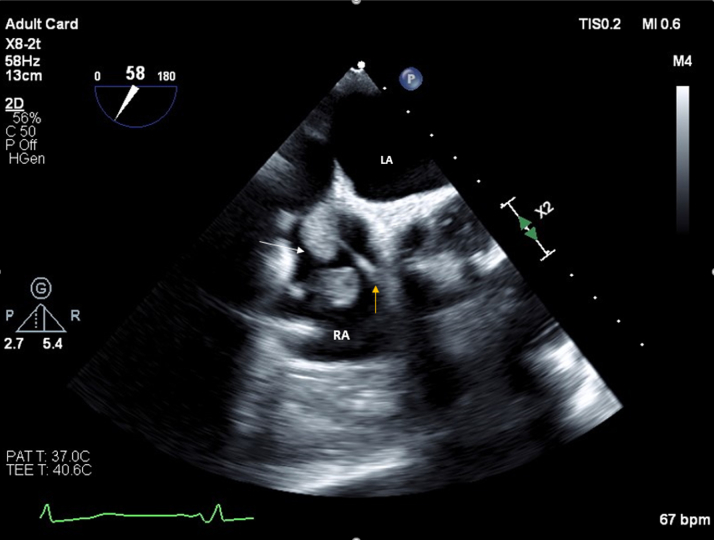
Transesophageal Echocardiography of Echodense Mass Transesophageal echocardiography revealed that the echodensity suspicious for thrombus/vegetation (white arrow) was attached to the right atrial lead (yellow arrow) and tricuspid annulus. LA = left atrium; RA = right atrium.

Blood tests revealed elevated inflammatory markers: C-reactive protein 104 mg/L and procalcitonin 0.47 μg/L. Blood cultures were positive for *Candida parapsilosis*. Transthoracic echocardiography (TTE) revealed a 4-cm mobile mass involving the tricuspid valve near the atrial pacing lead and attached to the posterior tricuspid leaflet; this mass was found to be prolapsing across the valve, with mild tricuspid regurgitation (Figures 1A and 1B). Transesophageal echocardiography (TEE) confirmed the large mass, suggestive of thrombus or vegetation, attached to the right atrial lead and tricuspid annulus (Figure 2).

## Differential Diagnosis

Given the presentation of fever, sepsis, and candidemia in the setting of the presence of new murmur and large mobile mass, the leading diagnosis was cardiac implantable electronic device (CIED)–related infective endocarditis (CIED-IE). Another possibility was a thrombus forming on the device lead with secondarily infection, but the echocardiographic characteristics were suggestive of vegetation rather than thrombus. Primary cardiac tumor was also possible, although less likely.

## Management

The patient was started on dual antifungal therapy (caspofungin and voriconazole) as well as cefixime by the infectious disease team. A multidisciplinary team including cardiology, electrophysiology, infectious disease, cardiac surgery, and vascular surgery reviewed the case. Open surgical repair was not feasible given the patient's high surgical risk. Although lead removal was deemed necessary to eliminate the source of the candidemia, the large size and mobility of the vegetation posed a high risk for fatal pulmonary embolization, making standalone device removal unfeasible. Consequently, the team opted for endovascular vegetation aspiration performed by vascular surgery, followed by CRT-D device and lead extraction by the electrophysiology team in a single procedure.

## Procedure

The patient was taken to the hybrid operating room, and the procedure was performed under general anesthesia. The right internal jugular vein (IJV) and right common femoral veins were accessed under ultrasound guidance in standard fashion, and 6-F sheaths were placed. Venogram was performed through the IJV sheath and showed that both brachiocephalic and superior vena cava veins were patent. Subsequently, a Terumo advantage wire was placed via the same access through the superior vena cava into the inferior vena cava with the support of an angled glide catheter (Terumo).

**Figure 3:**
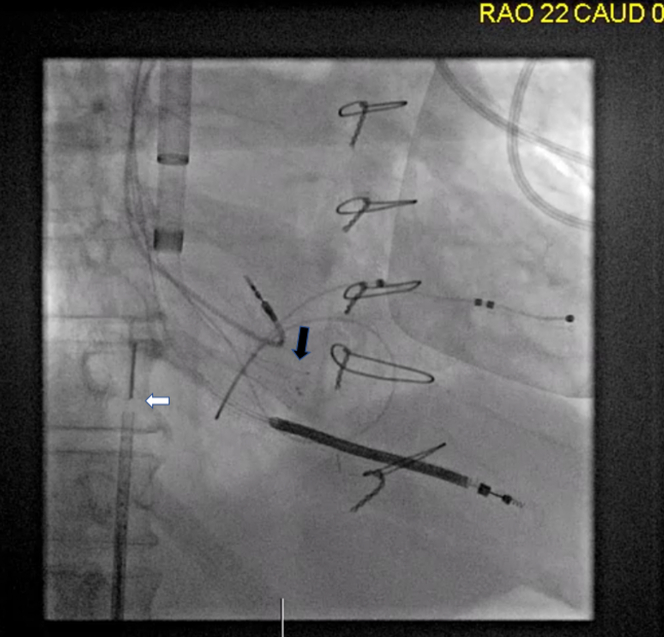
Fluoroscopic Image Demonstrating Catheter-Based Thrombus/Vegetation Aspiration The black arrow highlights the AlphaVac aspiration device positioned within the right atrium, and the white arrow indicates the intracardiac echocardiography catheter.

Initially, the wire was exchanged over the catheter for an Amplatz Super Stiff wire (Boston Scientific). Using the Amplatz wire, the IJV puncture site was sequentially dilated, and a 22-F DrySeal sheath (Gore Medical) was placed. The wire was then retracted and guided into the right ventricle using a glide catheter. After removal of the glide catheter, an 18-F AlphaVac thrombectomy system (Angiodynamics) was advanced under fluoroscopic guidance and positioned in the right atrium (Figure 3).

**Figure 4:**
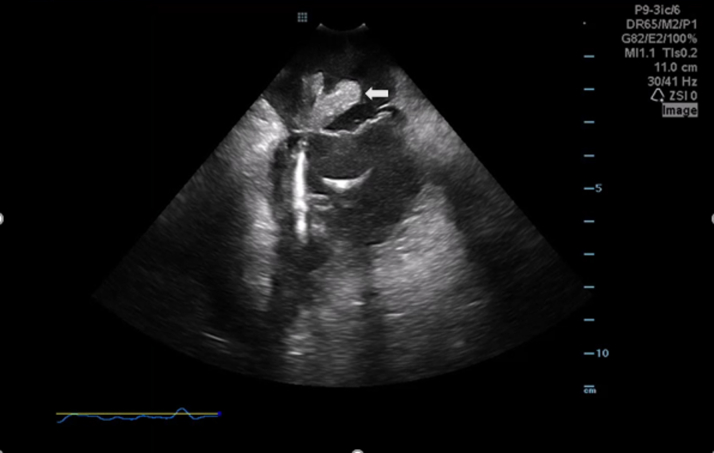
Intracardiac Echocardiography Demonstrating Large, Mobile Echodense Mass Intracardiac echocardiography clearly showed a large mass in the right atrium (arrow), consistent with vegetation.

Via right femoral access, an intracardiac echocardiography (ICE) catheter was advanced into the right atrium. The ViewFlex Xtra ICE catheter (Abbott) was used by the ICE operator, primarily owing to their extensive experience with using this catheter during electrophysiology procedures as part of a zero-fluoroscopy workflow; another factor in choosing this specific catheter was its excellent two-dimensional spatial resolution, which allowed for accurate identification of the vegetation site (Figure 4, Video 1). Under TEE guidance, the AlphaVac catheter was positioned to achieve the closest contact with the vegetation. Aspiration was then performed, successfully retrieving the vegetation on the first pass. The retrieved material was sent for microbiology and pathology analysis, and ICE confirmed the successful removal of most of the vegetation (Video 2).

Subsequently, the aspiration catheter was exchanged for a pigtail catheter (Boston Scientific), and a pulmonary angiogram was performed to assess for procedure-related pulmonary embolism; no embolism was found. After reversing heparin, the electrophysiology team proceeded with an uneventful extraction of the lead and the CIED.

Pathology revealed fibrinous vegetation with focal organization, containing numerous polymorphonuclear leukocytes and histiocytes. GMS (Gomori methenamine silver) and PAS (periodic acid–Schiff) stains demonstrated abundant colonies of fungal yeasts and hyphal elements within the vegetation, morphologically consistent with *Candida* species. The patient's condition improved within 48 hours after the procedure, with resolution of fever and improvement in inflammatory markers.

**Figure 5:**
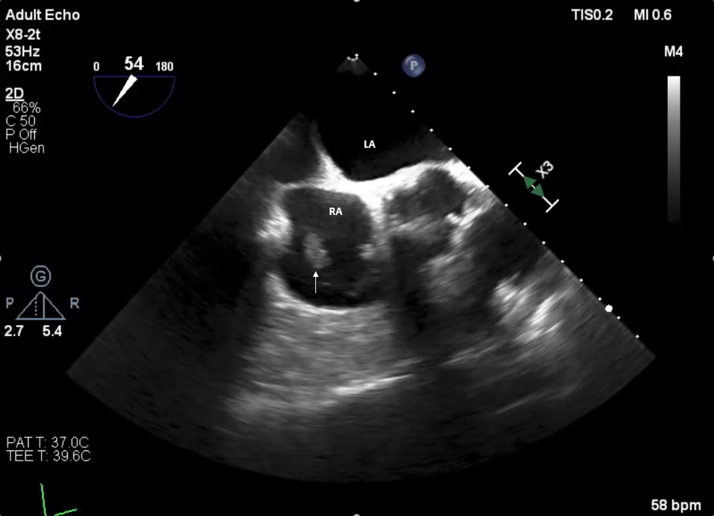
Transesophageal Echocardiography Within 6 Hours After Procedure Transesophageal echocardiography showing significant decrease in the number and size of tricuspid vegetation (arrow). LA = left atrium; RA = right atrium.

**Figure 6:**
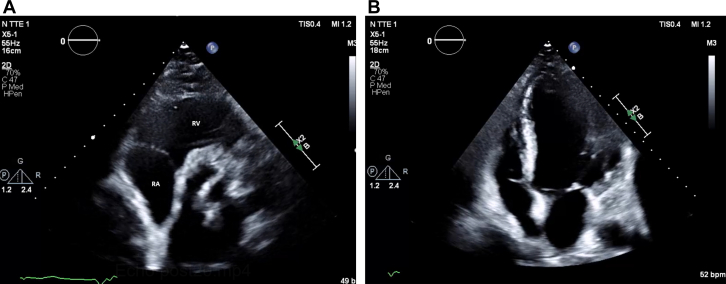
Echocardiography at 3-Month Follow-Up (A) Short-axis parasternal view and (B) apical 4-chamber view revealed complete resolution of vegetation. RA = right atrium; RV = right ventricle.

TEE within 6 hours showed a small residual vegetation on the tricuspid valve (Figure 5), prompting continuation of dual antifungals (caspofungin and voriconazole) for 6 weeks. At the 3-month follow-up, the patient remained afebrile, with normalized inflammatory markers (including C-reactive protein) and negative cultures. A repeat echocardiogram confirmed complete vegetation resolution (Figures 6A and 6B).

## Discussion

This report describes a challenging case of CIED-IE in a 69-year-old man with a prior CRT-D implantation. The patient presented with fever and candidemia but no signs of pocket involvement. TTE revealed a large, mobile 4-cm vegetation attached to the tricuspid valve and pacemaker lead.

### Diagnostic challenges and imaging considerations

The diagnosis of CIED-IE can be particularly challenging when patients exhibit elevated inflammatory markers and positive blood cultures without overt pocket infection. Although TTE and TEE are critical tools as per the 2019 European Heart Rhythm Association guidelines,^1^ they may not always clearly distinguish infected vegetations from noninfected thrombi. In our patient, the combination of significant vegetation size, positive candidemia, and TEE findings confirmed the diagnosis of CIED-IE.

### Treatment strategies and guideline recommendations

According to the 2010 and 2024 American Heart Association guidelines, complete device removal is mandatory to reduce the risk of relapse, as retained hardware is associated with high rates of recurrent infection.^2^^,^^3^ Evidence also suggests that early extraction, ideally within 7 days of diagnosis, is linked to lower 1-year mortality compared with delayed removal.^4^ For vegetations <2 cm, transvenous lead extraction is typically safe and effective, carrying a low risk of clinically significant pulmonary embolism. However, large vegetations (>2 cm) present a heightened risk of embolic complications. In such cases, some guidelines recommend that transcatheter vegetation debridement before transvenous lead extraction, or even surgical removal, should be considered.^5^ Special attention is needed for patients with anatomic predispositions, such as a patent foramen ovale or atrial septal defect, which can increase the risk of systemic embolization.^2^ These recommendations highlight the need for an individualized approach that balances the risks of embolization with the morbidity associated with surgical interventions. Surgical removal could also be considered in the setting of large lead vegetations; it is associated with greater morbidity but may reduce the risk of pulmonary embolism in select cases.^1^ Our patient had a 4-cm vegetation, which would have imposed very high risk for massive pulmonary embolism. Removal of such a large vegetation usually requires open surgery.

### Innovative endovascular technique

Given the prohibitive risks associated with open surgery in our patient, we employed a fluoroscopy-assisted, ICE-guided aspiration technique using the AlphaVac as an adjunct to conventional device extraction. This innovative approach used the high-resolution imaging capabilities of ICE combined with the real-time visualization of fluoroscopy, enabling precise debulking of the large vegetation. More extensive detail with ICE imaging during interventional and electrophysiology procedures can be achieved using three- and four-dimensional ICE imaging, although this modality was not available for the operators of our procedure. However, these advances in ICE imaging can significantly improve the visualization of complex intracardiac structures such as vegetations and enhance the operator's understanding of their relationship to the surrounding anatomical structures.

By reducing the vegetation mass before lead extraction, this method aims to reduce the risk of embolization while facilitating safer device removal. Although the literature supports the promise of this technique, its use remains largely limited to select cases, and standardized protocols have yet to be established.^6^ Further research is essential to confirm its effectiveness in reducing septic emboli and to assess long-term outcomes regarding infection control and device salvage.

## Conclusions

Managing CIED-IE in the presence of large vegetations and complicating factors such as candidemia is inherently complex. Adhering to established American Heart Association guidelines by ensuring early complete device removal is critical but carries serious risks. For patients who are not candidates for traditional surgical approaches, fluoroscopy-assisted, ICE-guided aspiration represents a promising, minimally invasive alternative that can reduce embolic risk and enhance procedural safety. The success observed in our case highlights the importance of a multidisciplinary approach, combining expertise from cardiology, vascular surgery, and infectious disease to individualize patient care. Future studies are warranted to refine these innovative techniques, establish standardized protocols, and evaluate long-term patient outcomes.

## Funding Support and Author Disclosures

The authors have reported that they have no relationships relevant to the contents of this paper to disclose.

## Appendix

Video 1 Intracardiac Echocardiography Revealing the Vegetation

Video 2 Intracardiac Echocardiography After Successful Removal of the Vegetation
